# Synchronous Primary Parosteal Osteosarcoma and Primary Mediastinal Germ Cell Tumour with Atypical Mycobacterial Infection – A Rare Phenomenon: A Case Report

**DOI:** 10.5704/MOJ.2303.023

**Published:** 2023-03

**Authors:** CH Lim, NH Mohamed-Haflah, MH Abdullah-Sani, CK Loh, MR Abdul-Rahman

**Affiliations:** 1 Department of Orthopaedic and Traumatology, Universiti Kebangsaan Malaysia, Kuala Lumpur, Malaysia; 2 Department of Paediatrics, Universiti Kebangsaan Malaysia, Kuala Lumpur, Malaysia; 3 Department of Surgery, Universiti Kebangsaan Malaysia, Kuala Lumpur, Malaysia

**Keywords:** germ cell tumour, mediastinal, parosteal osteosarcoma, synchronous, tuberculosis

## Abstract

Mediastinal germ cell tumours are a rare group of extragonadal germ cell tumours with less than 5% prevalence of all germ cell tumours. Primary mediastinal germ cell tumours themselves account for 16-36% of the extragonadal germ cell tumours. Along the spectrum of osteosarcoma, parosteal osteosarcoma is a well-differentiated surface osteosarcoma with a prevalence of 4% of all osteosarcoma. As such synchronous primary parosteal osteosarcoma and primary mediastinal germ cell tumour are exceedingly rare. This leads to complexity in determining the most appropriate chemotherapy for two different types of tumours and its potential side effects of reduced immunity leading to potential secondary infection. Here we report a case of a 16-year-old boy who presented with synchronous primary osteosarcoma and primary mediastinal germ cell tumour, complicated with atypical mycobacterial infection post-operatively. Additionally, we discuss our choice of chemotherapy and the management of the atypical mycobacterial infection.

## Introduction

Synchronous tumours are described as two or more distinct primary tumours diagnosed within six months of the initial diagnosis of the initial primary tumour. Parosteal osteosarcoma is a well-differentiated osteosarcoma with a good prognosis. Unlike classical intramedullary osteosarcoma, no chemotherapy is usually required, and curative treatment involves wide excision of the tumour^1^. A mediastinal seminoma is a rare group of extragonadal germ cell tumours that account for less than 5% of all germ cell malignancies^2^. Germ cells tumours in childhood, if ever present with secondary osteosarcoma, usually occurs later. Here, we report a case of primary parosteal osteosarcoma and primary mediastinal germ cell tumour, which has occurred synchronously. This case was further complicated with an anterior mediastinum atypical mycobacterial infection, which was detected post-operatively.

## Case Report

**Fig. 1:**
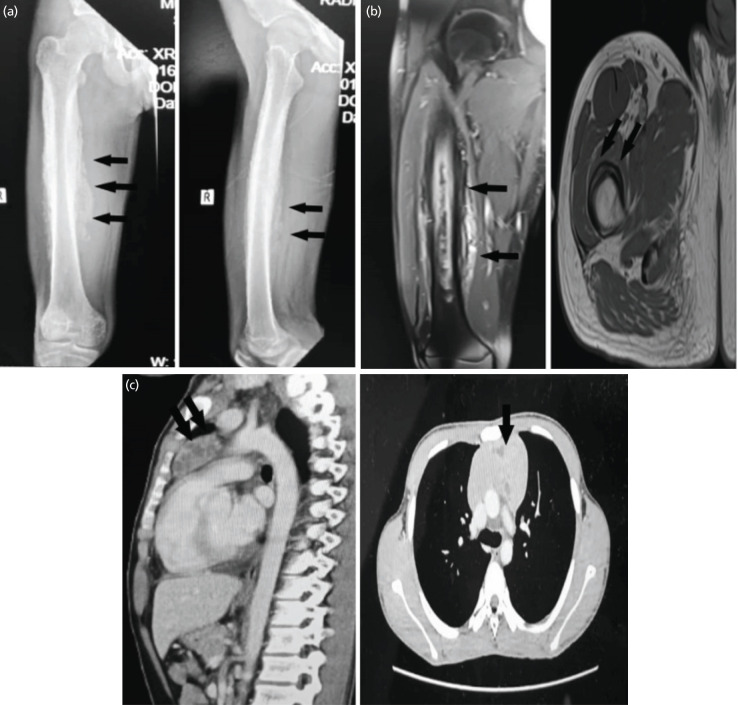
(a) Radiograph showing lamellated periosteal reaction along the diaphysis of the femur (arrow). (b) MRI showing features of the malignant bone tumour (arrow). (c) CT Thorax showing anterior mediastinal soft tissue mass (arrow).

A 16-year-old boy presented to us with a 9-month history of pain and swelling of the right thigh, associated with nocturnal pain and constitutional symptoms of weight loss and poor appetite. Clinical examination revealed a bony mass measuring 9x10cm extending from the proximal to the distal right thigh. Radiograph of the right femur showed lamellated periosteal reaction along the entire length of the diaphysis with increased soft tissue shadowing (Fig. 1a). MRI demonstrated features that were consistent with a malignant bone tumour with extraosseous soft tissue extension (Fig. 1b). Core needle biopsy with bone tissue samples confirmed a low-grade bone-forming tumour, likely parosteal osteosarcoma, with positive MDM2 amplification test.

Upon staging, his chest radiograph showed an anterior mediastinal mass. CT of the thorax demonstrated an anterior lobulated soft tissue mass measuring 5x7x9cm with intralesional hypodense area, which could be a cystic or necrotic component (Fig. 1c). The initial assumption was that of pulmonary metastasis. However, a biopsy of the mediastinal mass showed a germ cell tumour with histological evidence of seminoma. Additional tests were done to confirm mediastinal germ cell tumour. It was positive for CD 117, CKAE1/AE3 (perinuclear dot-like pattern) and negative for CD 20 and CD 3. Biochemical parameters of alpha-fetoprotein (AFP), lactate dehydrogenase (LDH) and erythrocyte sedimentation rate (ESR) were all raised. A significantly raised AFP according to his age (606.54ng/ml) led to the diagnosis of a malignant mixed germ cell tumour. The ultrasound of his testis, however, was normal.

The strategy of our treatment at this point was to address his mediastinum germ cell tumour with chemotherapy, followed by resection of both mediastinal germ cell tumour and parosteal osteosarcoma of the femur. He received multi-agent chemotherapy consisting of bleomycin, etoposide and cisplatin (BEP) according to the CCLG (Children’s Cancer and Leukaemia Group) Germ Cell Tumour Protocol. Repeated MRI and CT Thorax scan after three cycles showed a good response (>90% necrosis rate) with a significant reduction in the size of both the anterior mediastinal (with a residual volume of 9.1% as compared to pre-treatment volume) as well as the right thigh mass (with a residual volume of 26.2% as compared to pre-treatment volume). This was interesting as the chemotherapy intended for the mediastinal germ cell tumour affected the parosteal osteosarcoma too. He received a total of four cycles of chemotherapy before surgery.

**Fig. 2:**
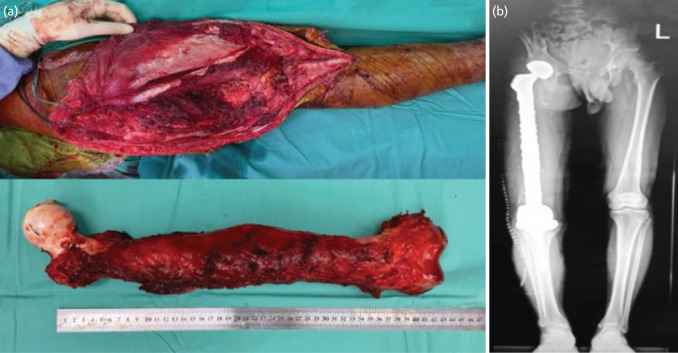
(a) Intra-operative picture post total femur resection. (b) Scanogram after total femur megaprosthesis.

Wide local excision of both tumours was carried out in the same setting. Open mini-sternotomy with excision of the mediastinal germ cell tumour was performed first. A video-assisted thoracoscopy was not performed in order to ensure good visualisation and, thus, achieved satisfactory tumour clearance. The second part of the surgery was wide local excision of parosteal osteosarcoma of the femur, which required removal of the entire femur (Fig. 2a). Skeletal reconstruction was achieved with the insertion of a total femur megaprosthesis (Fig. 2b).

**Fig. 3:**
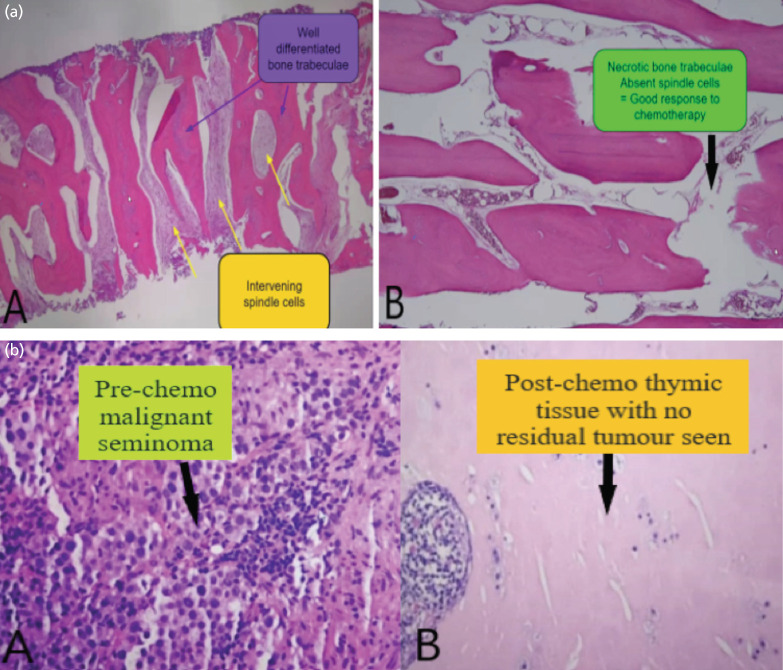
(a) Specimen of the femur, A (pre-chemotherapy); B (post-chemotherapy). (b) Specimen of anterior mediastinal mass, A (pre-chemotherapy); B (post-chemotherapy).

Histopathology of the resected femur showed post-treatment changes composed of necrotic neoplastic bone with no viable residual tumour seen with a good margin (Fig. 3a). Although the histology of the resected anterior mediastinal mass showed no residual tumour, it revealed the presence of a caseating granulomatous inflammation (Fig. 3b). The anterior mediastinal tissue cultures later yield mycobacterium species. However, the multiplex real-time polymerase chain reaction (PCR) was positive for non-tuberculous mycobacterial infection in the anterior mediastinum. Hence, he was treated for atypical mycobacterium infection and started on anti-tuberculous treatment of rifampicin, isoniazid, ethambutol, pyrazinamide and pyridoxine for a month. As he did not have any chest symptoms, the anti-tuberculous medications were discontinued. Due to the complete resolution of both the primary tumours, no further chemotherapy was given post-operatively.

At one year follow-up, he was able to ambulate without support. There was a limb length discrepancy of 2cm, which was compensated with a shoe raise. He had a good range of motion of both his right hip and knee. There was no evidence of local recurrence or distant metastasis.

## Discussion

Among all primary malignant bone tumours, osteosarcoma is the most common, with a prevalence of 19.2%^1^. Along the spectrum of osteosarcoma, parosteal osteosarcoma is a well-differentiated surface osteosarcoma accounting for 4% of all osteosarcoma^3^. It has a better prognosis than conventional osteosarcoma unless dedifferentiation has occurred. With a slight female predominance, it is more common in the third decade of life^3^. Common sites include the posterior aspect of the femoral metaphysis, followed by the proximal tibia and proximal humerus metaphysis. In cases where there is no evidence of distant metastasis, parosteal osteosarcoma carries a much better prognosis than classical high-grade osteosarcoma with a five-year disease-free survival rate of 90%^4^. Treatment in these cases involves wide excision of the tumour without neoadjuvant chemotherapy. Those with lung metastasis have a poorer prognosis, and current chemotherapy offers little benefit, with a degree of tumour necrosis rate and disease survival being unclear. However, several authors do recommend chemotherapy in cases of the dedifferentiated type and presence of lung metastasis^3^. The survival rate post-chemotherapy in these group of patients were reported to be close to 80%^5^. Our patient presented with a circumferential tumour involving almost the entire length of the femoral bone, with neurovascular involvement and soft tissue extension. Due to the extensive tumour involvement, our initial plan was for neoadjuvant chemotherapy with doxorubicin, cisplatin and methotrexate, followed by limb salvage surgery. However, the detection of a synchronous mediastinal germ cell tumour required an alternative approach to his management.

Mediastinal germ cell tumours are a rare group of extragonadal germ cell tumours with <5% prevalence of all germ cell tumours^2^. Primary mediastinal germ cell tumours themselves account for 16-36% of extragonadal germ cell tumours^2^. It may be divided into two groups histologically, namely seminomas and non-seminomas. Seminomas usually do not produce AFP. In our patient, a significantly raised AFP signify the presence of a malignant component, where the biopsy sample may not be representative of the whole tumour. Germ cell tumours mainly arise within the testis and are developmental cancers; however, they can also arise from extragonadal midline structures aside from the testis. One common hypothesis is that these tumour cells migrate along the genital ridge and survived in the extragonadal environment^2^. This patient presented with primary mediastinal germ cell tumour with no evidence of testicular germ cell tumours. Previous studies have demonstrated that genetic factors such as Li-Fraumeni syndrome, Klinefelter syndrome (KS) and retinoblastoma have an increased risk of developing these cancers^2^. However, the familial history of this patient did not appear to fit the clinical diagnostic criteria of a genetic predisposition for cancers such as Li-Fraumeni syndrome, Klinefelter syndrome and retinoblastoma. This child had a normal male chromosome and no clinical features of KS. The common presenting complaint of mediastinal germ cell tumours would be cough, haemoptysis, weight loss, fatigue and superior vena cava syndrome. However, our patient did not complain of any chest symptoms, hence an atypical presentation of mediastinal germ cell tumours.

The cure rate of histologically pure mediastinal seminomas could reach up to 90%^2^. In our case, the raised AFP suggested the presence of non-seminoma mixed with seminoma component. Non-seminomas, however, have a poorer prognosis despite cisplatin-based chemotherapy and thoracic surgery^2^. The conventional treatment includes chemotherapy with bleomycin, etoposide and cisplatin (BEP) with surgical excision, with a success rate of 86%^9^. Herein lies the complexity in the management of our patient with respect to determining the chemotherapy regime for two different primary tumours. Combining both regimes or administering it consecutively was not advocated due to the overwhelming toxicities and potential drug resistance. Compared to parosteal osteosarcoma, the aggressive nature of the mediastinal malignant mixed germ cell tumour prompted us to give priority to the latter with respect to our choice of chemotherapy regime. Moreover, we anticipated that the cisplatin-based chemotherapy may have an effect on the femur lesion and facilitate the limb salvage surgery. Our decision to administer BEP chemotherapy had shown good outcomes for both tumours.

Advancement in chemotherapy has led to improved survival in patients with malignant tumours and the possibility of performing limb salvage surgery. Despite this, it is not without its complications. For surgeons, immunological compromise can lead to a delay of surgery, further prolonging the treatment. Post-operatively, secondary infection can impede wound healing and have disastrous consequences when a prosthesis is involved. Tuberculous infection was detected in our patient through histological examination of the anterior mediastinal lesion. It is possible that the infection was contracted prior to chemotherapy. The patient displayed constitutional symptoms of weight loss and anorexia, but this was assumed to be due to the malignancy. There were no respiratory symptoms or other risk factors. According to our national clinical practice guidelines on tuberculosis management, based on many years of well-designed randomised controlled trials (RCT), the treatment protocol consists of two months daily dose of rifampicin, isoniazid, ethambutol, pyrazinamide and pyridoxine (intensive phase); four months daily dose of rifampicin and isoniazid (maintenance phase). Whenever possible, for the whole duration of treatment, rifampicin should be used. Unfavourable outcomes are significantly higher in regimens without rifampicin compared to those with rifampicin in the maintenance phase. The optimal course of treatment is at least six months. Here, however, we only treated him with anti-tuberculous medications for a month considering the side effects of the medications as well as the fact that he did not exhibit any chest symptoms.

The occurrence of three different diagnoses in the same patient is rare, requiring us to modify the patient's treatment in order to achieve a high cure rate with minimal complications. This case is a reminder to tumour surgeons to always be aware of the possibilities of simultaneous primary malignant tumours as this could potentially affect treatment modalities and prognosis of the patient. Long-term follow-up is also crucial, especially with young survivors of cancers, even after successful treatment for primary cancers.
